# Dynamics of histone H2A, H4 and HS1ph during spermatogenesis with a focus on chromatin condensation and maturity of spermatozoa

**DOI:** 10.1038/srep25089

**Published:** 2016-04-28

**Authors:** Zhao-Hui Zhang, Shu-Mei Mu, Ming-Shen Guo, Jiang-li Wu, Yan-qin Li, Han Zhang, Ying Wang, Xian-Jiang Kang

**Affiliations:** 1 College of Life Sciences, Hebei University, Baoding 071000, China; 2 Department of Reproductive Medicine, Maternal and Child Care Service Centre, Baoding 071000, China

## Abstract

Histones and histone phosphorylation play vital roles during animal spermatogenesis and spermatozoa maturation. The dynamic distribution of histones H2A and H4 and phosphorylated H2A and H4 at serine 1 (HS1ph) was explored in mammalian and Decapoda germ cells, with a special focus on the distribution of H2A, H4 and HS1ph between mouse condensed spermatozoa chromatin and crab non-condensed spermatozoa chromatin. The distribution of histone marks was also analysed in mature spermatozoa with different chromatin structures. Histone H2A and H4 marks were closely associated with the relatively loose chromatin structure in crab spermatozoa. The significant decrease in the HS1ph signal during spermatogenesis suggests that eliminating most of these epigenetic marks in the nucleusis closely associated with spermatozoa maturity.

Chromatin architecture and function are regulated by complex mechanisms involving histone members, histone variants, remodelling complexes and post-translational histone modifications^1^,^2^. Histone phosphorylation is involved in many chromatin-related events, including transcriptional regulation, double-strandbreak (DSB) repair and sub-nuclear organization. Histone phosphorylation can directly alter the nucleosome surface to affect chromatin organization and higher-orderstructure, to create new docking interfaces and to recruit specific protein complexes. Histone phosphorylation at serine residues is generally associated with gene activation and regulates transcription factor accessibility to chromatin, thus affecting gene transcription^3^,^4^,^5^,^6^,^7^ and chromatin condensation, which might occur independently or cooperatively^8^,^9^.

Chromatin condensation and decondensation are important processes during the cell cycle^1^. Phosphorylation of histone H3 at serine 10 and threonine 3 is thought to be necessary for these processes during mitosis^10^,^11^. Phosphorylation of H4 serine 1 (H4S1ph) [Fig. 1(a)] and H2B serine 10 has been implicated in this process in yeast^10^,^11^,^12^. H4S1ph is also thought to be part of the DNA repair histone code and is one of the common post-translational modifications (PTMs) that occur during spermatogenesis^13^. H4S1ph and H2A serine 1 phosphorylation [Fig. 1(a)] is specifically localized to double-strand breaks (DSBs) *in vivo* and might be closely related to DSB recombination and meiotic chromosome condensation^14^,^15^. H4S1ph is also regarded as a prerequisite modification for the highly orchestrated process of histone replacement^16^ and is thought to be linked to transcription through the regulation of nuclear volume, chromatin compaction, and DNA accessibility^17^,^18^.

Chromatin condensation also plays a vital role in spermatozoa maturity. In mature human and mouse spermatozoa, the nucleus becomes smaller and denser and takes on a characteristic flattened form. The significant change in nuclear content (termed nuclear condensation) reduces the size of the nucleus to a large extent. For example, in *Xenopus*, spermatozoa DNA condenses through interactions with a protein complex containing one molecule each of histone H3, histone H4, and the sperm-specific proteins X and Y^19^. Crabs (*Eriocheir sinensis*) are representative members of phylum Arthropoda, subphylum Crustacea, order Decapoda. Crab spermatozoa are globular in shape and are composed of the central acrosome vesicle, cytoplasm and nucleus. The central acrosome vesicle is surrounded by cytoplasm and a cup-shaped nucleus with no flagellum [Fig. 1(b)]. The acrosome is penetrated in the centre by the perforatorium. The nucleus contains slightly condensed chromatin, which is relatively looser than that in mouse spermatozoa [Fig. 1(b)]; thus, it is classified as a non-condensed nucleus^20^,^21^. The details of the structural composition and function of non-condensed chromatin remain unclear. A recent study showed that acetylated histone H4 was present at the centre of the nucleus and was accompanied by chromatin fibres in the crustacean shrimp *Fenneropenaeus chinensis*, in which the nucleus of spermatozoa is also in a loose state similar to that in *E.* sinensis^22^.

There are few sensitive markers for identifying normal spermatozoa, especially for *in vitro* fertilization (IVF) or intra-cytoplasmic injection (ICSI). Because histones and histone phosphorylation are essential for chromatin condensation, we designed this study to evaluate the dynamic distribution of H2A, H4 and H2A/H4 phosphorylated at serine 1 (HS1ph) in testis germ cells from two different animals (mouse and crab). Using both longitudinal (during spermatogenesis) and cross-sectional (between mice and crabs) approaches, we aimed to explore the dynamics and distributions of these three markers during spermatogenesis, with a special focus on their distribution in different forms of spermatozoa chromatin (condensed or non-condensed) [Fig. 1(b)] and different stages of spermatozoa maturation.

## Results

**Figure 1:**
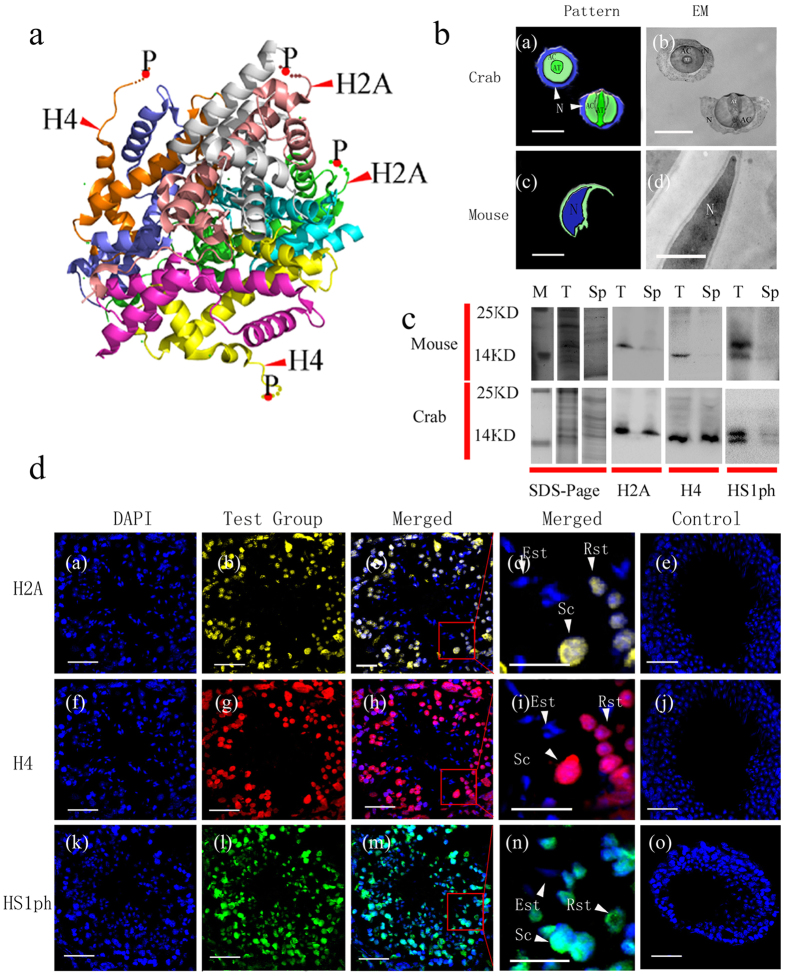
(**a**) Histone octamer with H2A, H4 and HS1ph. (**b**) Condensed and non-condensed chromatin of crab and mouse spermatozoa; (**c**) cropped and stitched image of SDS-PAGE and Western blot analysis of H2A, H4 and HS1ph; (**d**) immunofluorescence analysis of H2A and H4 in mouse testes. (**a**) N, nucleus; AT, acrosomaltubule; AC, acrosomalvesicle, P, phosphorylated serine 1 of histone H2A (or H4). Bar = 5 μm. Histone octamerthree-dimensional structure (see the supplementary file for further details) (PDB code 1TZY) showing histones H2A and H4 and the phosphorylated serine 1(P) residues. (**b**)[**b-**(a)] Crab spermatozoa nuclei are doughnut-shaped in the cross-sectional view and cup-shaped in the vertical view. [**b-**(b)] Non-condensed chromatin with a loose structure in the nuclei stained light grey in an immunoelectron microscopy image. [**b-**(c)] Sickle-shaped nuclei in mice. [**b-**(d)] Condensed chromatin stained dark black in electron microscopy images. (**c**) M: marker; T: testes protein; Sp: spermatozoa protein. Total protein was extracted,electrophoresed under the same experimental conditions via SDS-PAGE and analysed by Western blotting. Positive immunoblotting for histone H2A, H4 and HS1ph was present in the Test groups for both species. Histone H2A and H4 were present in Sp samples from crabs, but not those from mice. HS1ph was absent in Sp samples from both animals. (**d**) Sc (spermatocytes), Rst (round spermatids), Est (elongated spermatids). Scale bar = 20 μm. Fluorescent images of DAPI (blue), H2A (yellow), H4(red) and HS1ph (green)staining of mouse germ cell sections. [**d** (a,f,k)] DAPI-stained nuclei in cross-sections of mouse seminiferous tubules. [**d** (b,g,l)] H2A, H4 and HS1ph staining of these cross-sections. Merged images of a and b (c), f and g (h), and k and l (m) (c,h,m). Magnified sections of images c, h and m indicating Sc, Rst and Est (d,i,n). [**d** (b–d,g–i)]Positive staining for total H2A and H4 was observed mostly in the nuclei of Sc and Rst. [**d** (l–n)] Positive staining for HS1ph was found in both the cytoplasm and nuclei of mouse Sc and Rst, but not in Est nuclei. [**d** (e,j,o)] Merged images of DAPI staining and the negative fluorescent control for the primary antibody. No H2A, H4 or HS1ph staining was observed.

Western blot analysis revealed that the antibodies employed in this study specifically reacted with proteins of a certain molecular weight without nonspecific binding [Fig. 1(c)]. The mouse and crab germ cells were classified and identified based on cell size, the nuclear-to-cytoplasmic ratio and DNA morphology using immunofluorescence and immunoelectron microscopy. Spermatocytes(Sc) are round with a diameter greater than 10 μm. The key features of Sc are a high nuclear-to-cytoplasmic ratio and highly condensed DNA. Round spermatids (Rst) are characterized by adiameter of approximately 5 μm, a high nuclear-to-cytoplasmic ratio and highly condensed DNA. Elongated spermatids (Est) are oval or round and are the same size as Rst; they are characterized by elongated nuclei. The nuclei of Est are crescent- or doughnut-shaped in crabs and sickle-shaped in mice. Chromatin condensation has already begun in Est. Pachytene Sc, Rst, Est and mature spermatozoa represent the typical stages of spermatogenesis.

Immunofluorescence was performed on both testis sections and cell smears. The former revealed the distribution of the marks in germ cells in different stages in the original testes visually and globally, and the latter more clearly showed the marks in each representative germ cell without the interference of fluorescence from the adjacent tissue (or cells).

**Figure 2 f2:**
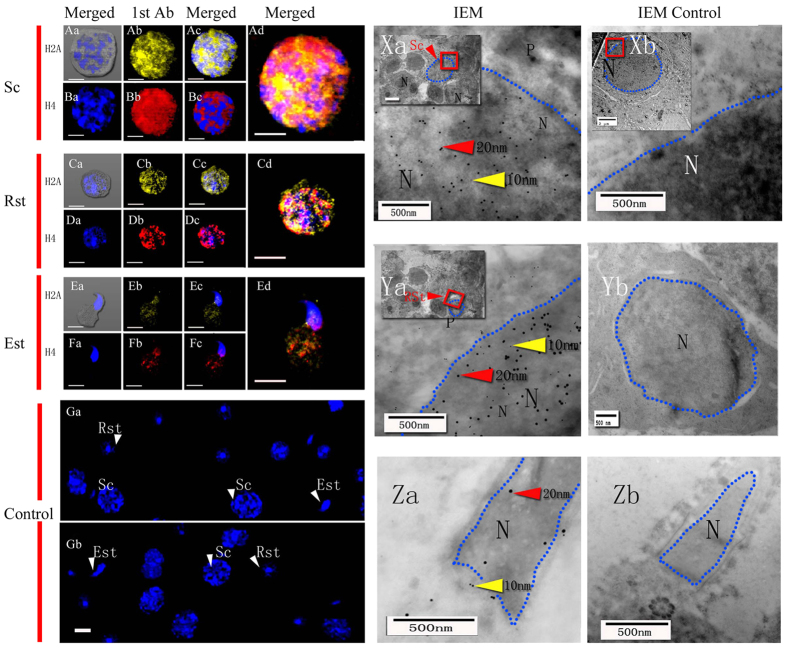
Immunofluorescence and immunoelectron microscopy detection of H2A and H4 distribution in mouse germ cells. Sc (spermatocytes), Rst (round spermatids), Est (elongated spermatids), 1st Ab (primary antibody), IEM (immunoelectron microscopy), N (nucleus). Scale bar (immunofluorescence, IF) = 20 μm; Scale bar (IEM) = 500 nm. Fluorescent images of DAPI (blue), H2A (yellow) and H4(red) staining. Blue dotted lines indicate the boundary of the nuclear area. Positive staining for total histone H2A was observed in the nuclei and cytoplasm of Sc (Aa-d), Rst(Ca-d) and Est(Ea-d). Positive staining for total histone H4 was found in the nuclei and cytoplasm of Sc (Ba-d), Rst (Da-d) and Est (Fa-d). (Ga and Gb), Negative staining for total histone H2A and H4 was observed in the control. Colloidal gold-labelled H2A (10 nm, yellow arrows) and H4 (20 nm, red arrows) were located in the cytoplasm and nuclei of Sc(Xa), Rst(Ya) and EST(Za). Colloidal gold was mainly located in the nuclei of germ cells. (Xb, Yb and Zb) Individual scale bars are shown in each panel. No colloidal gold staining was observed for total histone H2A and H4 in the control group.

**Figure 3 f3:**
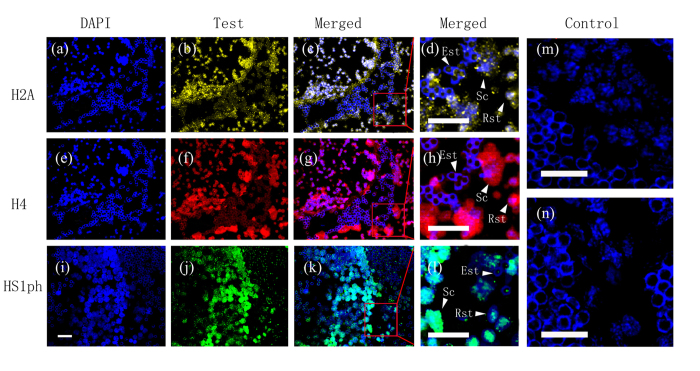
Immunofluorescence detection of H2A and H4 distribution in crab testes. Sc (spermatocytes), Rst (round spermatids), Est(elongated spermatids). Scale bar = 20 μm. Fluorescent images of DAPI (blue), H2A (yellow), H4(red) and HS1ph (green) staining of crab germ cell sections. (**a**,**e**,**i**) DAPI-stained nuclei of cross-sections of the crab seminiferous tubule. (**b**,**f**,**j**) H2A, H4 and HS1ph staining of the cross-sections. (**c**,**g**,**k**) Merged images of **a** and **b** (**c**), **e** and **f** (**g**), and **i** and **j** (**k**). (**d**,**h**,**l**) Magnified sections from images **c**, **h** and **m** indicating the widely distributed marks in Sc, Rst and Est. (**b–d, f–h**) Positive staining for total histone H2A and H4 was found mostly in the nuclei of Sc and Rst. (**j–l**) Positive staining for HS1ph was found in both the cytoplasm and nuclei of mouse Sc and Rst, but not in Est nuclei. (**m,n**) No H2A, H4 or HS1ph staining was observed in the controls.

**Figure 4 f4:**
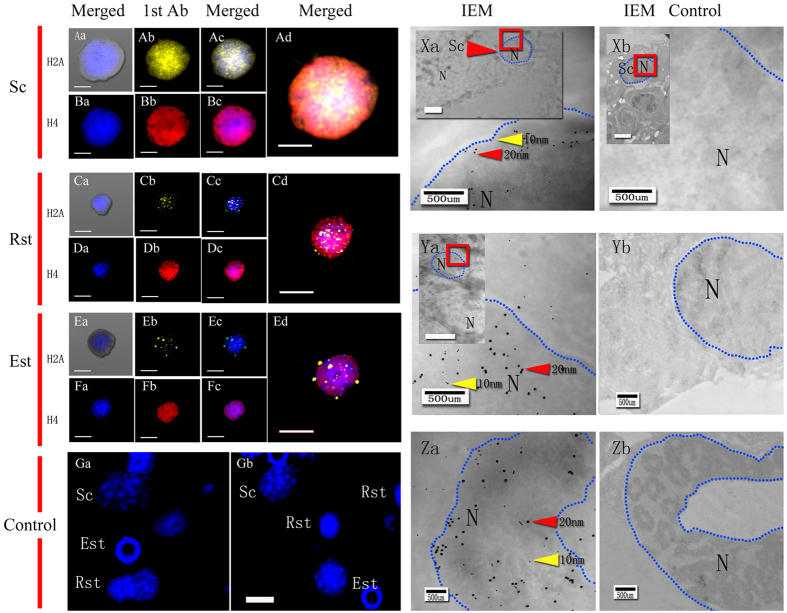
Immunofluorescenceand immunoelectron microscopy detection of H2A and H4 distribution in crab germ cells. Sc (spermatocytes), Rst (round spermatids), Est (elongated spermatids), 1st Ab (primary antibody), IEM (immunoelectron microscopy), N (nucleus). Scale bar (immunofluorescence, IF) = 20 μm; scale bar (IEM) = 500 nm. Fluorescent images of DAPI (blue), H2A (yellow) and H4 (red) staining. Positive staining for total histone H2A was found in the nuclei and cytoplasm of Sc (Aa-d), Rst (Ca-d) and Est (Ea-d). Positive staining for total histone H4 was observed in the nuclei and cytoplasm of Sc (Ba-d), Rst (Da-d) and Est (Fa-d). Negative staining for total histone H2A and H4 was observed in the controls (Ga and Gb). Colloidal gold-labelled H2A (10 nm, yellow arrows) and H4 (20 nm, red arrows) staining was observed in the cytoplasm and nuclei of Sc (Xa), Rst (Ya) and EST (Za). Blue dotted lines indicate the boundary of the nuclear area. Colloidal gold was mainly located in the nuclei of germ cells. Individual scale bars are shown in each panel. No colloidal gold staining for total histone H2A or H4 was observed in the control group (Xb, Yb and Zb).

**Figure 5:**
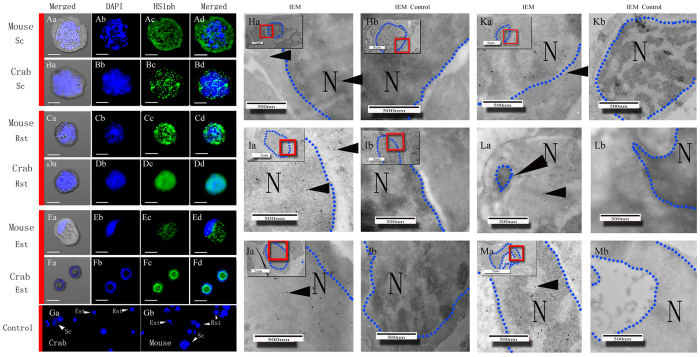
Immunofluorescence and immunoelectron microscopy detection of the distribution of HS1ph in mouse and crab spermatozoa. Sc (spermatocytes), Rst (round spermatids), Est (elongated spermatids), 1st Ab (primaryantibody), IEM (immunoelectron microscopy), N (nucleus). Scale bar (immunofluorescence, IF) = 20 μm; scale bar (IEM, picture-in-picture) = 5 μm; scale bar (IEM) = 500 nm. Fluorescent images of DAPI (blue) and HS1ph (green). Blue dotted lines indicate the boundary of the nuclear area. Positive staining for HS1ph was distributed in the nuclei and cytoplasm of Sc (mouse: Aa-d; crab: Ba-d), Rst (mouse: Ca-d; crab, Da-d) and Est(mouse: Ea-d; crab, Fa-d). Positive staining for HS1ph was observed in both the nuclei and cytoplasm of Sc and Est. The staining appears weaker in Est nuclei, mainly being located in the cytoplasm around the nucleus. IEM images show the distribution of gold particles in the nuclei and cytoplasm of Sc (mouse: Ha; crab: La), Rst (mouse: Ja; crab: Ka) and, to a lesser extent, Est (mouse: La; crab: Ma) with elongated spermatids. Black arrows indicate the gold particles. No gold particles were observed in the control germ cells (mouse: Hb, Jb, Lb; crab: Ib, Kb, Mb). There were no green (HS1ph) immunofluorescence signals in the control Sc, Rst and Est (mouse:Gb; crab: Ga).

Immunofluorescence staining and immunoelectron microscopy images revealed that H2A, H4 and HS1ph were localized (100 ± 0%) in the nuclei of Sc, Rst, and Est from mice and *E. sinensis* as well as in plasma [Fig f2][Fig f3][Fig f4](Figs 2–5).

**Figure 6:**
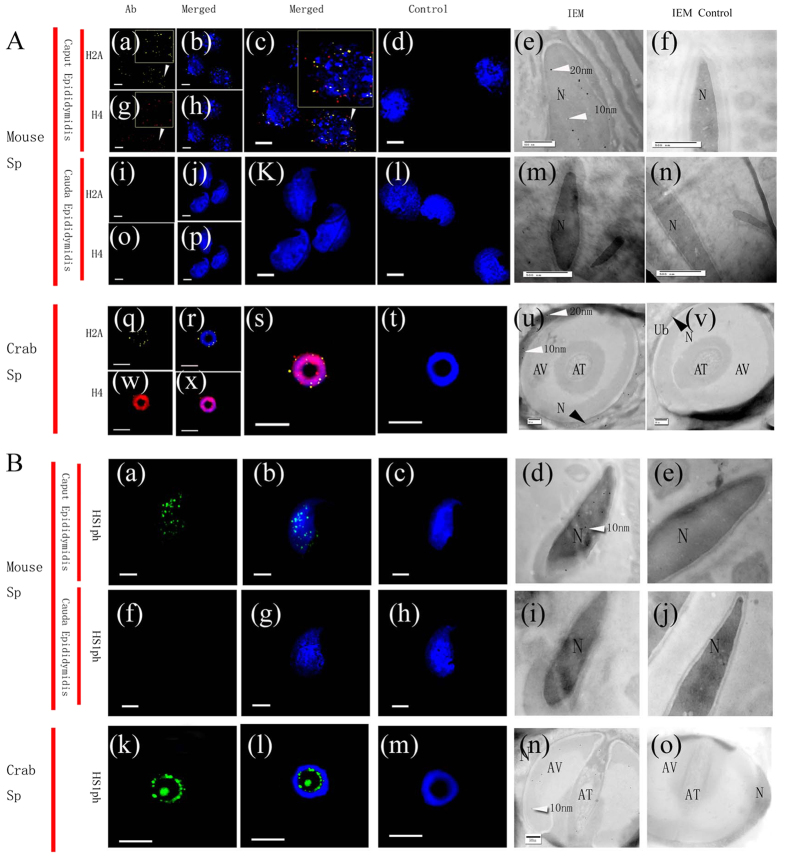
Immunofluorescence and immunoelectron microscopy detection of the distribution of H2A, H4 (**A**) and HS1ph (**B**) in mouse and crab spermatozoa. Sc (spermatocytes), Rst (round spermatids), Est(elongated spermatids), Ab (primary antibody), IEM (immunoelectron microscopy), N (nucleus). Scale bar (immunofluorescence, IF) = 20 μm; scale bar (IEM) = 500 nm. Fluorescent images of DAPI (blue), H2A (yellow) and H4(red) staining. (**A**) There was positive staining for H2A and H4 in decondensed nuclei (a–c,g,h) but little or no staining in decondensed cauda epididymis Sp nuclei (i–l,o,p). No signals were observed in the control group (d). Both 10-nm (H2A) and 20-nm (H4) gold particles were observed in Sp nuclei in the caput epididymis of mice(e,m), but few or no gold particles were found in Sp nuclei in the cauda epididymis (m). Both 10-nm (H2A) and 20-nm (H4) gold particles were observed in the nuclei of crab Sp (u). No gold particles were observed in the nuclei of the control group (f,n,v). (**B**) There was positive staining for HS1ph in decondensed caput epididymis Sp nuclei (a,b), but little or no staining in decondensed cauda epididymis Sp nuclei (f,g). Ten-nanometre (HS1ph) gold particles were observed in the nuclei of caput epididymis Sp (e). There were few or no gold particles in the nuclei of cauda epididymis Sp in mice (m). Positive staining for HS1ph was observed in the AC and AT of crab spermatozoa (n). No signals were observed in the control group (c,j,o).

**Figure 7:**
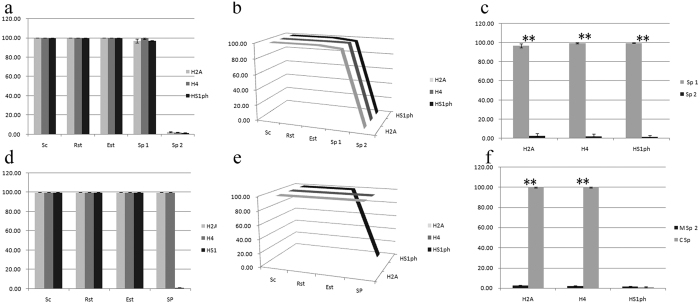
Statistical analysis of the positive staining rate (%) for H2A, H4 and HS1ph in germ cell nuclei. Sc (spermatocytes), Rst (round spermatids), Est (elongated spermatids), cauda epididymis spermatozoon (Sp1), cauda epididymis spermatozoon (Sp2), nucleus-positive rate (NPR), **(P < 0.001). The NPRs for the three markers in mouse germ cells are shown (**a,b**). The NPR for the three markers was 100% in mouse Sc, Rst and Est. These markers showed a slight decrease in Sp1 and significantly decreased in Sp2 (**a,b**). The differences in the NPRs for the three markers were significant between Sp1 and Sp2 (P < 0.001; **c**). The results for the three markers in crab germ cells are shown in (**d**,**e**); the NPR of the histone H2A and H4 markers was 100% in crab Sc, Rst, Est and Sp. The NPR of the HS1ph mark paralleled that of the other two markers in crab Sc, Rst and Est but decreased significantly in Sp (**d,e**). When the NPRs of the spermatozoa of the two animals were compared, the H2A and H4 markers were significantly different (P < 0.001; (**f**)), but there was no difference in HS1ph (P > 0.05; **f**).

Mouse caput epididymis spermatozoa (Sp1) were positive for H2A (97 ± 2.12%), H4 (99.50 ± 0.76%) and HS1ph (97 ± 0.38%) [Figs 6(A) and 7], but the expression of these markers was significantly decreased (H2A, 2.44 ± 0.68%; H4, 2.06 ± 0.46%; and HS1ph, 1.44 ± 0.62%) in spermatozoa from the cauda epididymis (Sp2) [Figs 6(A) and 7]. Statistical analysis showed that the nucleus-positive rates (NPRs) of these three markers were significantly different between spermatozoa from the caput epididymis and cauda epididymis (P < 0.001) (Fig. 7).

Opposite distributions of histones H2A and H4 were observed in the mature spermatozoa of mice and crabs [Figs 6(A) and 7]. The NPR (%) for histones H2A and H4 was 100 ± 0% in crabs, versus 2.44 ± 0.68% and 2.06 ± 0.46%, respectively, in mice [Figs 6(A) and 7]. The HS1ph marker exhibited a contrasting result compared with the other markers, with an NPR of 1.44 ± 0.62% observed in mice and 1.19 ± 0.37% in crabs (P = 0.418) [Figs 6(B) and 7].

## Discussion

In the present study, we choose pachytene Sc, Rst, Est and mature spermatozoa to represent the typical stages of spermatogenesis. The pachytene spermatocyte phase begins after the pairing of homologous chromosomes. As spermatogenesis progresses, there is widespread reorganization of the haploid genome, followed by extensive DNA compaction in Rst^23^,^24^,^25^. Histones H2A, H4 and HS1ph were localized in the nucleus and cytoplasm of Sc (Figs 2 and 4), which is consistent with reports indicating that H2A and H4 synthesis proceeds during this period^24^,^26^. In our work, male germ cells from the two species were observed to undergo different series of morphological transformations during spermatogenesis to generate typically shaped spermatozoa^20^,^21^,^27^,^28^,^29^,^30^,^31^. The similar distributions of the three markers started to change when the elongation of spermatids began. Unlike the H2A and H4 markers, HS1ph decreased with the elongation of mouse and crab spermatids, despite the presence of super-condensed DNA in mouse sperm and non-condensed DNA in crab sperm. This finding suggests that HS1ph has different functions compared with histone H2A and H4 during spermatozoa DNA compaction. The present study revealed the dynamics of the three markers specifically and visually during spermatogenesis in two specific species and suggested that mice and crabs exhibit the same HS1ph dynamics during spermatogenesis; thus, this mark may have a significant impact during meiosis, regardless of sperm morphology and chromatin status.

Spermatozoa are highly specialized cells in which nearly all transcriptional events are arrested. These cells can serve as an ideal model for studying the role of histones and histone modification in chromatin architecture. Mouse spermatozoa adopt a flattened shape with highly compact, condensed chromatin, which may help optimize the nuclear shape and support the ability of the gametes to swim through the female reproductive tract to the oocytes^32^. Super-compaction of the genome is also thought to provide additional protection from genotoxic factors^33^. Replacement of nucleosomes with protamines is frequently observed in mammals^23^,^25^,^28^,^34^,^35^. In both mouse and human sperm, histones are localized to the nucleus^26^. However, only approximately 1% of the histones in mouse spermatozoa are reserved in the nucleosome, compared with approximately 15% in human spermatozoa^36^,^37^,^38^. The degree of this chromatin substitution is known to vary among species. According to our experiments, minimal amounts of H2A (2.06%) and H4 (6.09%) were observed in condensed spermatozoa chromatin.In contrast to the findings in mice, the non-condensed chromatin in crab spermatozoa is thought to be necessary for nuclear deformation during fertilization. Research on the basicproteins present in sperm nuclei has included few species of Decapoda. Previous reports have indicated that there are nearly no histones or protamines in the sperm nuclei of decapod crustaceans^39^,^40^,^41^,^42^,^43^,^44^,^45^,^46^. However, recent studies have demonstrated the presence of histones in decapod sperm nuclei^47^,^48^,^49^. A recent study from our laboratory confirmed the presence of histones H3 and H4 in the nuclei of *E. sinensis* spermatozoa^49^. In the present study, H2A and H4 were visualized in spermatozoa nuclei with loose chromatin, whereas few histones were observed in nuclei with condensed chromatin. This study confirmed that the conservation of both H2A and H4 is closely related to the loose status of crab chromatin. The newly identified mode of DNA compaction in Decapoda, beyond the elementary structural unit of non-condensed chromatin, is interesting and must be further studied.

It is well established that most changes in histones are crucial, as they may alter gene expression patterns or cause other abnormalities^50^. For example, staining of spermatozoa with aniline blue, which shows the degree of histone conservation, has been performed in infertile men to evaluate the degree of chromatin compaction. It has been reported that considerable nuclear instability in sperm and a high percentage of stained spermatozoa are detectable in infertile groups^51^,^52^. As the nuclear contents are not only inheritable but also essential for fertilization, our discovery that H2A and H4 were conserved in the nuclei of mature mouse spermatozoa (albeit at low concentrations) suggested that a certain amount of H2A and H4 conservation in nuclei may represent more accurate marks of mature mouse spermatozoa. Detection of H2A, H4 and H4S1ph may help to determine the aetiology of infertility as well as identify appropriate treatments.

Epigenetics refers to the study of heritable changes that do not involve alteration of DNA. The paternal genome, with certain associated epigenetic messages, contributes to the growing embryo, where it plays important roles in early developmental processes and influences newborn offspring^53^. It has been reported that the timing and localization of HS1ph during mitosis are similar and are closely correlated with chromatin condensation events during mitosis; thus, this mark was suggested to play a dual role in chromatin condensation during mitosis and histone deposition in S-phase^14^, and H2A serine 1 phosphorylation has been reported to be necessary for proper entry into the histone chaperone pathway in the early embryo^54^. In the mitotic chromatin of worms, flies, and mammals, H4S1ph was observed to persist until relatively late in gametogenesis^17^. However, few specific roles for these two widely distributed histone modifications have been demonstrated. In the present study, HS1ph was found to persist in spermatocytes (Sc) and round spermatids (Rst); when the DNA began to condense in elongated spermatids (Est), the HS1ph mark decreased in the condensed mouse DNA and the non-condensed crab DNA. To be more precise, characteristic HS1ph dynamics in the nucleus and cytoplasm synchronized the maturation process of spermatozoa, which was confirmed by two findings from this study. First, the HS1ph marks were significantly decreased during spermiogenesis, and certain HS1ph marks were reserved in mature spermatozoa nuclei in both mice and crabs. Second, HS1ph marks were significantly decreased in mouse spermatozoa nuclei during the transition. Previous reports have documented that spermatozoa do not undergo active transcription and translation. Therefore, extra-testicular spermatozoa maturation is not regulated by the germinal genome; rather, it is regulated by certain factors in epididymal fluid^55^,^56^,^57^,^58^. The fact that the use of spermatozoa derived from the testes and caput epididymis always leads to a lower fertilization rate in IVF than the use of spermatozoa from the cauda epididymis also suggests that some biological event occurs in the epididymis to promote the super-compaction of mature spermatozoa chromatin^55^,^56^,^57^,^58^. Thus, one of the obvious changes observed during the super-compaction of mature spermatozoa was the disappearance of most HS1ph marks, which implies that this mark is not only closely associated with spermatozoa chromatin architecture but is also correlated with the arrest of active biological events. These results are consistent with previous studies^17^. Based on the data reported herein, we propose that a certain percentage of HS1ph is reserved in the nucleus as a marker of mammalian spermatozoa maturation. We have designed controlled studies on normal and premature crabs as well as on normal and pathological sperm from patients undergoing ART treatment. The results and conclusions are greatly anticipated.

We suggest that there is a close relationship between histones H2A and H4 and the relatively loose state of crab spermatozoa chromatin. The significant decrease in the HS1ph signal during spermatozoa maturation observed in both mice and crabs suggests a close relationship between spermatozoa maturity and the loss of most of the HS1ph epigenetic markers in the nucleus.

## Materials and Methods

### Animals

Adult male ICR mice at 4–6 weeks of age (n = 8) were selected after mating with female mice that subsequently gave birth to live newborns. Sexually mature male crabs (n = 8) were selected in this study. The protocol was approved by the Committee on the Ethics of Animal Experiments of Hebei University. All procedures used in the animal experiments were compliant with the local approved protocols of the Administration Office Committee for Laboratory Animals, and all efforts were made to minimize animal suffering.

### Preparation of testes and spermatozoa

The mice were anesthetized with sodium pentobarbital. After skin disinfection (with 75% alcohol), an incision was made in the skin and peritoneum. After pushing away the bowels and fat and forcing the testes out of the scrotum (into abdomen), the testes and epididymis were surgically removed. The caput epididymis and cauda epididymis were cut into blocks and mechanically disrupted using a syringe (1 ml) under an anatomical microscope in Quinn’s Sperm Washing Medium (Cat. No. 1006, Quins’ SAGE IVF, USA) at 37 °C. Spermatozoa were obtained from the surface of the cell suspension after incubation at 37 °C for 20 min. The crabs were anesthetized by chilling on ice for 15 min. The carapaces were cut open cross-sectionally, and the heart and accessory sex gland were removed gently. Subsequently, the testes and seminal vesicles were exposed and surgically removed. To obtain free spermatozoa from crabs, we dissected the seminal vesicles into pieces, and spermatozoa were obtained via manual tissue homogenization in Ca^2+^-free artificial seawater (Ca2^+^-FASW; 475 mM NaCl, 12 mM KCl, 30 mM MgCl_2_, 20 mM Tris, pH 8.2). After being left to stand for 20 min at 4 °C, the free spermatozoa were collected by centrifugation (10,000 rpm for 10 min, supernatants reserved; 500 rpm for10 min, supernatants reserved and 1000 rpm for 10 min, pellet collected).

### Western blot analysis

The testes were dissected and homogenized. Then, the homogenate (or spermatozoa pellet) was resuspended in Phosphosafe Extraction Reagent (Cat. No. 71296-3, Novagen) and centrifuged (12,000 rpm, 5 min, 0–4 °C). Protein concentrations were determined with the Bradford Protein Assay (Bio-Rad), and the protein samples were used in SDS-PAGE (15% SDS-PAGE, 120 V, 0–4 °C) and analysed by Western blot. Western blots were performed according to standard procedures. Primary antibodies were used at the following dilutions: anti-phosphorylated H2A and H4 serine 1 antibody (1:4000, Cat. No. ab14723; Abcam) with Halt Phosphatase Inhibitor Cocktail; anti-H2A antibody (1:1000, Cat. No. ab177308; Abcam); and anti-H4 antibody (1:1000, Cat. No. ab31830; Abcam). The following secondary antibodies were used: HRP-labelled goat anti-rabbit IgG (H + L) (1:200, Cat. No. A0208; Beyotime) and HRP-labelled goat anti-mouse IgG (H + L) (1:200, Cat. No. A0216; Beyotime).

### Immunofluorescence analysis

Immunofluorescence was performed on both testis sections and cell smears. Testes from male mice and crabs were fixed in 4% paraformaldehyde (PFA) in Tris-buffered saline (TBS; 140 mM NaCl and 20 mM Tris-HCl, pH 7.6) for 48 h, dehydrated in 30% sucrose and embedded in Optimal Cutting Temperature(OCT) formulation (Sakura Finetek Japan Co., Ltd). Frozen sections were cut at 5-μm thickness and placed on lysine-coated glass slides. Smears were made on poly-D-lysine-coated slides. The germ cell smears were prepared after digesting fresh tissue with Cell Dissociation Buffer (0.05% tryptase, 37 °C, 15 min; Cat. No. 13150-016, Thermo Fisher Scientific). The condensed mouse spermatozoa nuclei were decondensed with a decondensing mix (25 mM DTT, 0.2% Triton X-100, and 200 IU/ml heparin; Leo Laboratories) for 15 min; the reaction was complete when most nuclei exhibited twice the surface area of non-decondensed sperm heads. Slides of the germ cells (from testes) or spermatozoa were made and fixed in 4% formaldehyde for 15 min. The slides were then washed with TBS for 15 min, treated with Quick Antigen Retrieval Solution (Cat. No. P0090, Beyotime) for 5 min at RT, permeabilized in TBS with 0.5% saponin and 0.25% Triton X-100 for 30 min and then rinsed three times (10 min/wash) in TBST (TBS and 0.1% Tween-20). The slides were next blocked in TBST with 5% BSA, 0.1% TritonX-100, and 6% goat serum for 2 h at 37 °C and then incubated with primary antibody in TBS with 5% BSA, 0.1% TritonX-100, and 6% goat serum for 48 h at 4 °C. The anti-HS1ph (with 1% Halt Phosphatase Inhibitor Cocktail), anti-H4 and anti-H2A primary antibodies were used at 1:500, 1:100 and 1:100 dilutions, respectively. After the samples were incubated with the primary antibodies, they were washed three times (15 min/wash) with TBST at RT and then incubated with an Alexa Fluor^®^ 488-conjugated goat anti-rabbit (1:300 dilution, Cat. No. ab150097; Abcam) or DyLight^®^ 550-conjugated goat anti-mouse (1:300 dilution, Cat. No. ab98737; Abcam) secondary antibody in TBS with 5% BSA at 37 °C for 1 h. DAPI (1:10,000 dilution, Cat. No. H-1200; Vector Lab) was used to stain the cell nuclei. Digital images were obtained using a confocal laser microscopesystem (Olympus FV1000-IX81; numerical aperture 90–300 μm; excitation wave lengths 405 nm, 488 nm, and 543 nm; CA = 400 μm and HV = 400V for testis sections; CA = 100 μm and HV = 400V for cell smears) and image processing software (FV10-ASW 1.7 Viewer and Photoshop 7.0). Two hundred representative germ cells per mouse or crab were observed via fluorescence microscopy (FV10-ASW 1.7), and the percentage of positive staining for each primary antibody was recorded. All images of a given cell were obtained with the same gain factor. Negative controls were generated by omitting the primary antibody and were detected strictly using the same confocal laser microscope and the same gain factor.

### Immunoelectron microscopy analysis

The testes from 8 mice and 8 crabs were dissected quickly at 0–4 °C. The samples were then fixed in 4% formaldehyde and 0.5% glutaraldehyde in TBS for 4 h at 0–4 °C. After dehydration in ethanol (30%, 50%, 70%, 85%, and 95% ethanol for 10 min, followed by three changes of 100% ethanol for 10 min each), the samples were transferred to a 2:1 mixture of hard-grade LR white resin (London Resin Co., Ltd., UK) and 100% ethanol. Finally, the sample blocks were embedded in LR white resin following the standard procedure. Ultra-thin (70-nm thickness) slices were prepared with a Reichert-Jung 701704 Ultra cut Cutter Ultra-Microtome, and the sections were mounted on a 200-mesh gold TEM grid. The sections were subsequently blocked in TBS with 1% BSA for 2 h at 37 °C and then incubated with anti-H2A and anti-H4 antibodies (both at a 1:40 dilution) or with an anti-HS1ph antibody (1:80 dilution) in TBS with 0.1% BSA overnight at 4 °C. Next, the sections were washed six times (10 min/wash) with TBST at RT and incubated with 10-nm colloidal gold-conjugated goat anti-rabbit IgG (H + L) (1:50 dilution, Cat. No. ab39601; Abcam) and 20-nm colloidal gold-conjugated goat anti-mouse IgG (H + L)(1:50 dilution, Cat. No. ab24272; Abcam) at RT for 1 h. The sample grids were then rinsed three times (10 min/wash) with TBS and three times (10 min/wash) with distilled water and subsequently stained with uranylacetate. Negative controls were generated by omitting the primary antibody. The sample grids were examined with a transmission electron microscope at 100 kV (JEM-100SX TEM, NEC, Tokyo, Japan).

### Statistical analysis

Positive staining in the nucleus, as observed by fluorescence microscopy, indicated positive cells. The nucleus-positive rate (NPR, %) was recorded and analysed. At least 200 cells of each specific germ cell type were counted from every animal. All statistical analyses were performed using SPSS v.18.0 (IBM Corp., Armonk, NY, USA). The data are presented as the mean ± SD. After numerical transformation of the NPR%, significant differences were assessed via one-way analysis of variance (ANOVA), and p values < 0.001 were considered significant.

## Additional Information

**How to cite this article**: Zhang, Z.-H. *et al.* Dynamics of histone H2A, H4 and HS1ph during spermatogenesis with a focus on chromatin condensation and maturity of spermatozoa. *Sci. Rep.* **6**, 25089; doi: 10.1038/srep25089 (2016).

## Supplementary Material

Supplementary Information

## References

[b1] Kouzarides T. Chromatin modifications and their function. Cell 128, 693–705 (2007). 17320507 10.1016/j.cell.2007.02.005

[b2] Ruchaud S., Carmena M. & Earnshaw W. C. Chromosomal passengers: conducting cell division. Nat. Rev. Mol. Cell Bio. 8, 798–812 (2007). 17848966 10.1038/nrm2257

[b3] Fingerman I. M., Wu C.-L., Wilson B. D. & Briggs S. D. Global loss of Set1-mediated H3 Lys4 trimethylation is associated with silencing defects in Saccharomyces cerevisiae. J. Biol. Chem. 280, 28761–28765 (2005). 15964832 10.1074/jbc.C500097200 PMC2745054

[b4] Shogren-Knaak M. Histone H4-K16 acetylation controls chromatin structure and protein interactions. Science 311, 844–847 (2006). 16469925 10.1126/science.1124000

[b5] Jenuwein T. & Allis C. D. Translating the histone code. Science 293, 1074–1080 (2001). 11498575 10.1126/science.1063127

[b6] Altaf M. *et al.* Interplay of chromatin modifiers on a short basic patch of histone H4 tail defines the boundary of telomeric heterochromatin. Mol. Cel. 28, 1002–1014 (2007). 10.1016/j.molcel.2007.12.002 PMC2610362 18158898

[b7] Berger S. L. Histone modifications in transcriptional regulation. Curr. Opin. Genet. Dev. 12, 142–148 (2002). 11893486 10.1016/s0959-437x(02)00279-4

[b8] Carrell D. T. & Hammoud S. S. The human sperm epigenome and its potential role in embryonic development. Mol. Hum. Reprod. 16, 37–47 (2010). 19906823 10.1093/molehr/gap090

[b9] Kang H., Park Y. S., Cho D.-H., Kim J.-S. & Oh J. S. Dynamics of histone H3 phosphorylation at threonine 3 during meiotic maturation in mouse oocytes. Biochem. Biophys. Res. Commun. 458, 280–286 (2015). 25645018 10.1016/j.bbrc.2015.01.099

[b10] Fischle W. *et al.* Regulation of HP1–chromatin binding by histone H3 methylation and phosphorylation. Nature 438, 1116–1122 (2005). 16222246 10.1038/nature04219

[b11] Dai J., Sultan S., Taylor S. S. & Higgins J. M. The kinase haspin is required for mitotic histone H3 Thr 3 phosphorylation and normal metaphase chromosome alignment. Genes Dev. 19, 472–488 (2005). 15681610 10.1101/gad.1267105 PMC548948

[b12] Ahn S.-H. *et al.* Sterile 20 kinase phosphorylates histone H2B at serine 10 during hydrogen peroxide-induced apoptosis in S. cerevisiae. Cell 120, 25–36 (2005). 15652479 10.1016/j.cell.2004.11.016

[b13] Sung M. T. & Dixon G. H. Modification of histones during spermiogenesis in trout: a molecular mechanism for altering histone binding to DNA. Proc. Acad. Nat. Sci. 67, 1616–1623 (1970). 10.1073/pnas.67.3.1616 PMC283398 5274484

[b14] Barber C. M. *et al.* The enhancement of histone H4 and H2A serine 1 phosphorylation during mitosis and S-phase is evolutionarily conserved. Chromosoma 112, 360–371 (2004). 15133681 10.1007/s00412-004-0281-9

[b15] Prigent C. & Dimitrov S. Phosphorylation of serine 10 in histone H3, what for? J. Cell Sci. 116, 3677–3685 (2003). 12917355 10.1242/jcs.00735

[b16] Wendt K. D. & Shilatifard A. Packing for the germy: the role of histone H4 Ser1 phosphorylation in chromatin compaction and germ cell development. Genes Dev. 20, 2487–2491 (2006). 16980578 10.1101/gad.1477706

[b17] Krishnamoorthy T. *et al.* Phosphorylation of histone H4 Ser1 regulates sporulation in yeast and is conserved in fly and mouse spermatogenesis. Genes Dev. 20, 2580–2592 (2006). 16980586 10.1101/gad.1457006 PMC1578680

[b18] Tessarz P. & Kouzarides T. Histone core modifications regulating nucleosome structure and dynamics. Nat. Rev. Mol. Cell Bio. 15, 703–708 (2014). 25315270 10.1038/nrm3890

[b19] Philpott A. & Leno G. H. Nucleoplasmin remodels sperm chromatin in Xenopus egg extracts. Cell 69, 759–767 (1992). 1591776 10.1016/0092-8674(92)90288-n

[b20] Nanshan D. & Luzheng X. Induction of acrosome reaction of spermatozoa in the decapodaEriocheir sinensis. Chin. J. Oceanol. Limnol. 5, 118–123 (1987).

[b21] Du Nanshan X. L. & Wei L. Studies on the sperm of Chinese mitten-handed crab, Eriocheir Sinensis (crustacea, decapoda) II. spermatogenesis [J]. Oceanol Limnol Sin 1, 008 (1988).

[b22] Ge S.-q., Kang X.-j., Guo M.-s. & Mu S. Immunolocalization of Basic Protein H4 During Spermatogenesis of Fenneropenaeus chinensis. Journal-Hebei university natural science edition 28, 187 (2008).

[b23] Wykes S. M. & Krawetz S. A. The structural organization of sperm chromatin. J. Biol. Chem. 278, 29471–29477 (2003). 12775710 10.1074/jbc.M304545200

[b24] Zentner G. E. & Henikoff S. Regulation of nucleosome dynamics by histone modifications. Nat. Struct. Mol. Biol. 20, 259–266 (2013). 23463310 10.1038/nsmb.2470

[b25] Yunes R., Doncel G. F. & Acosta A. A. Incidence of sperm-tail tyrosine phosphorylation and hyperactivated motility in normozoospermic and asthenozoospermic human sperm samples. Biocell-mendoza - 27, 29–36 (2003). 12847912

[b26] Cremer T. *et al.* Chromosome territories, interchromatin domain compartment, and nuclear matrix: an integrated view of the functional nuclear architecture. Crit. Rev. Eukaryot. Gene Expr. 10, 179 (2000). 11186332

[b27] Oliva R. & Dixon G. H. Vertebrate protamine genes and the histone-to-protamine replacement reaction. Prog. Nucleic Acid Res. Mol. Biol. 40, 25–94 (1991). 2031084 10.1016/s0079-6603(08)60839-9

[b28] Toshimori K. & Ito C. Formation and organization of the mammalian sperm head. Arch. Histol. Cytol. 66, 383–396 (2003). 15018141 10.1679/aohc.66.383

[b29] Bellve, A. R., Millette, C. J. O’Brien C. F., Bhatnagar D. A. Dym Y. M. M. Spermatogenic cells of the prepuberal mouse: Isolation and morphological characterization. J. Cell Bio. 74, 18 (1977). 10.1083/jcb.74.1.68 PMC2109873 874003

[b30] Kula K. The completion of spermatogenic cells in the course of spermatogenesis in immature rats. Folia Morphol. (Warsz.) 36, 167–173 (1977). 303205

[b31] Bate M. & Arias A. M. The development of Drosophila melanogaster. Vol. 1 (Cold Spring Harbor Laboratory Pr, 1993).

[b32] Moreno Díaz de la Espina S., Alverca E., Cuadrado A. & Franca S. Organization of the genome and gene expression in a nuclear environment lacking histones and nucleosomes: the amazing dinoflagellates. Eur. J. Cell Biol. 84, 137–149 (2005). 15819396 10.1016/j.ejcb.2005.01.002

[b33] Kota S. K. & Feil R. Epigenetic transitions in germ cell development and meiosis. Dev. Cell 19, 675–686 (2010). 21074718 10.1016/j.devcel.2010.10.009

[b34] Lewis J. D., Song Y., de Jong M. E., Bagha S. M. & Ausió J. A walk though vertebrate and invertebrate protamines. Chromosoma 111, 473–482 (2003). 12743711 10.1007/s00412-002-0226-0

[b35] Lewis J. D., Abbott D. W. & Ausió J. A haploid affair: core histone transitions during spermatogenesis. Biochem. Cell Biol. 81, 131–140 (2003). 12897846 10.1139/o03-045

[b36] Bench G., Friz A., Corzett M., Morse D. & Balhorn R. DNA and total protamine masses in individual sperm from fertile mammalian subjects. Cytometry 23, 263–271 (1996). 8900468 10.1002/(SICI)1097-0320(19960401)23:4<263::AID-CYTO1>3.0.CO;2-I

[b37] Tanphaichitr N., Sobhon P., Taluppeth N. & Chalermisarachai P. Basic nuclear proteins in testicular cells and ejaculated spermatozoa in man. Exp. Cell Res. 117, 347–356 (1978). 720415 10.1016/0014-4827(78)90148-9

[b38] Zalensky A. O., Breneman J. W., Zalenskaya I. A., Brinkley B. & Bradbury E. M. Organization of centromeres in the decondensed nuclei of mature human sperm. Chromosoma 102, 509–518 (1993). 8243163 10.1007/BF00368344

[b39] Jamieson B. G. Ultrastructural comparison of the spermatozoa of Ranina ranina (Oxystomata) and of other crabs exemplified by Portunus pelagicus (Brachygnatha)(Crustacea, Brachyura). Zoomorphology 109, 103–111 (1989).

[b40] Vaughn J. C. Changing nuclear histone patterns during development I. fertilization and early cleavage in the crab, Emerita Analoga. J. Histochem. Cytochem. 16, 473–479 (1968). 4878494 10.1177/16.7.473

[b41] Vaughn J. & Locy R. Changing nuclear histone patterns during development III. The deoxyribonucleic acid content of spermatogenic cells in the crab Emerita analoga. J. Histochem. Cytochem. 17, 591–600 (1969). 4186253 10.1177/17.9.591

[b42] Langreth D. C. Approximate screening functions in metals. Physical Review 181, 753 (1969).

[b43] Vaughn J. C. & Thomson L. A. A kinetic study of DNA and basic protein metabolism during spermatogenesis in the sand crab, Emerita analoga. J. Cell Bio. 52, 322–337 (1972). 4109924 10.1083/jcb.52.2.322 PMC2108633

[b44] Kleve M. G., Yudin A. I. & Clark W. H. Fine structure of the unistellate sperm of the shrimp, Sicyonia ingentis (Natantia). Tissue Cell 12, 29–45 (1980). 7361302 10.1016/0040-8166(80)90050-6

[b45] Kang X., Li S., Wang G. & Xiang Y. Distribution of basic proteins of sperm and fertilization in Scylla serrata. Dong wu xue bao.[Acta zoologica Sinica] 47, 82–86 (2000).

[b46] Kurtz K., Martínez-Soler F., Ausió J. & Chiva M. Histones and nucleosomes in Cancer sperm (Decapod: Crustacea) previously described as lacking basic DNA‐associated proteins: A new model of sperm chromatin. J. Cell. Biochem. 105, 574–584 (2008). 18655193 10.1002/jcb.21857

[b47] Kurtz K., Ausió J. & Chiva M. Preliminary study of sperm chromatin characteristics of the brachyuran crab Maja brachydactyla. Histones and nucleosome-like structures in decapod crustacean sperm nuclei previously described without SNBPs. Tissue Cell 41, 334–344 (2009). 19324386 10.1016/j.tice.2009.02.003

[b48] Stewart M. J. *et al.* Spermatogenesis in the blue swimming crab, Portunus pelagicus, and evidence for histones in mature sperm nuclei. Tissue Cell 42, 137–150 (2010). 20413138 10.1016/j.tice.2010.03.002

[b49] Wu J.-L., Kang X.-J., Guo M.-S., Mu S.-M. & Zhang Z.-H. Cloning and Functional Analysis of Histones H3 and H4 in Nuclear Shaping during Spermatogenesis of the Chinese Mitten Crab, Eriocheir sinensis. PLos One 10, 1–17 (2015). 10.1371/journal.pone.0126623 PMC4438001 25993499

[b50] Castillo J., Estanyol J. M., Ballescà J. L. & Oliva R. Human sperm chromatin epigenetic potential: genomics, proteomics, and male infertility. Asi. J. Andr. 17, 601–609 (2015). 10.4103/1008-682X.153302 PMC4492051 25926607

[b51] Molnar Z. *et al.* Sperm concentration, hyaluronic acid-binding capacity, aneuploidy and persistent histones in testicular cancer. Hum. Reprod. 29, 1866–1874 (2014). 25047166 10.1093/humrep/deu179

[b52] Rajender S., Avery K. & Agarwal A. Epigenetics, spermatogenesis and male infertility. Mutation Research/Reviews in Mutation Research 727, 62–71 (2011). 10.1016/j.mrrev.2011.04.002 21540125

[b53] Wang W.-L. *et al.* Developmental regulation of histone H2A deposition via serine-1 phosphorylation. Epigenet. Chromatin 6, P74 (2013).

[b54] Johnston D. S. *et al.* The mouse epididymal transcriptome: transcriptional profiling of segmental gene expression in the epididymis. Biol. Reprod. 73, 404–413 (2005). 15878890 10.1095/biolreprod.105.039719

[b55] Turner T. & Hinton B. The Third International Conference on the Epididymis. *Charlottesville, VA: The Van Doren Company* (2003).

[b56] Jervis K. M. & Robaire B. Dynamic changes in gene expression along the rat epididymis. Biol. Reprod. 65, 696–703 (2001). 11514330 10.1095/biolreprod65.3.696

[b57] Kirchhoff C. Gene expression in the epididymis. Int. Rev. Cytol. 188, 133–202 (1999). 10208012 10.1016/s0074-7696(08)61567-3

[b58] Toh G. W. Histone H2A phosphorylation and H3 methylation are required for a novel Rad9 DSB repair function following checkpoint activation. DNA Repair (Amst.) 5, 693–703 (2006). 16650810 10.1016/j.dnarep.2006.03.005

