# Supplementary material for: Dynamics of histone H2A, H4 and HS1ph during spermatogenesis with a focus on chromatin condensation and maturity of spermatozoa
Source: Sci Rep. 2016 Apr 28;6:25089. doi: 10.1038/srep25089 (PMC4848542; doi:10.1038/srep25089)
Supplement: Supplementary Information [file srep25089-s1.pdf]

Title: Dynamics of histone H2A, H4 and HS1ph during spermatogenesis with a focus on chromatin condensation and maturity of spermatozoa

Authors list: Zhao-Hui Zhang, Shu-Mei Mu, Ming-Shen Guo, Jiang-li Wu, Yan-qin Li, Han Zhang, Ying Wang, Xian-Jiang Kang

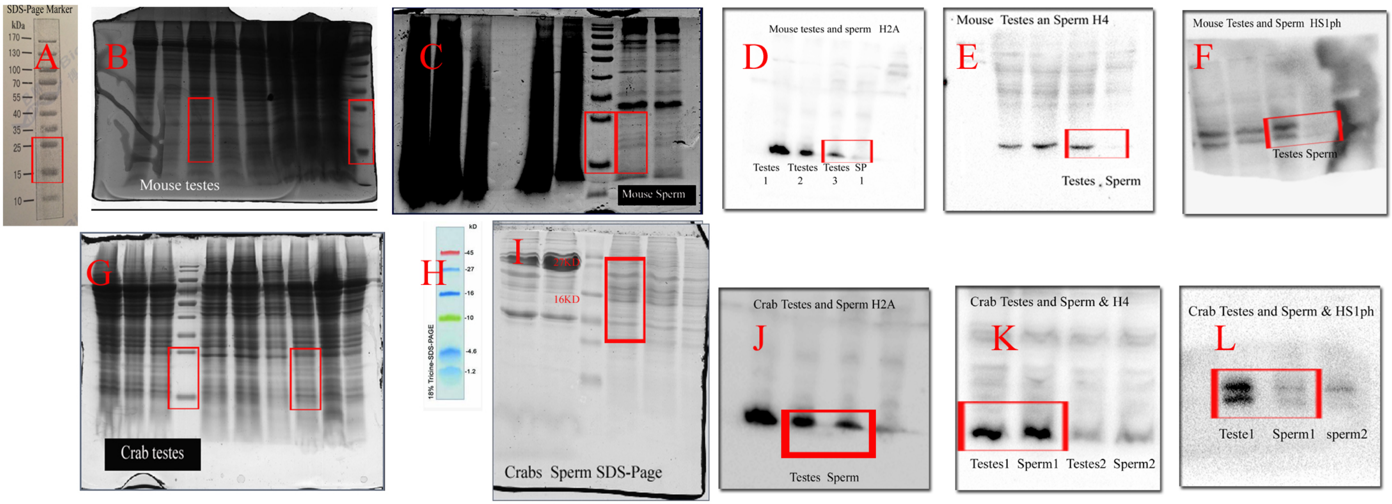

Original Figure SDS-Page & Blot

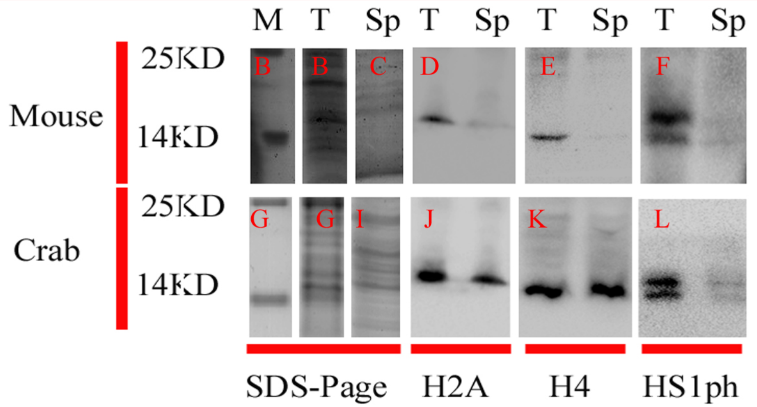

Cropped Gels and Blots in the paper

Images of SDS-Page Gels and Blots were cropped together into one image. The same capital letters indicate the original and the cropped images.
